# Effects of Preoperative Antiplatelet Agents and Anticoagulants on Total Joint Arthroplasty Outcomes

**DOI:** 10.31486/toj.20.0169

**Published:** 2021

**Authors:** Gonzalo Sumarriva, Alexander Habashy, Tara Saxena, George Chimento

**Affiliations:** ^1^ Department of Orthopedic Surgery, Ochsner Clinic Foundation, New Orleans, LA; ^2^ The University of Queensland Faculty of Medicine, Ochsner Clinic Foundation, New Orleans, LA; ^3^ Department of Orthopedic Surgery, Louisiana State University–Shreveport, Shreveport, LA

**Keywords:** *Anticoagulants*, *arthroplasty*, *hip*, *intraoperative complications*, *knee*

## Abstract

**Background:** Postoperative total joint arthroplasty complications place a tremendous burden on the health care system. The purpose of this study was to compare 30-day postoperative complication rates for surgeries in patients who received preoperative antiplatelet agents and/or anticoagulants to surgeries in a control group that did not receive antiplatelet agents and/or anticoagulants in the 90 days prior to undergoing a total joint arthroplasty.

**Methods:** We retrospectively reviewed total hip or knee arthroplasties from November 2012 to March 2016. Surgeries were categorized into 4 groups depending on their preoperative antiplatelet and anticoagulant status. Complications between the groups were compared using chi-square analysis and Fisher exact test.

**Results:** In this study, 1,726 arthroplasties in 1,544 patients were included. Superficial wound complications were the most common complication in all 4 groups (3.8% of surgeries), with no significant difference between the groups. A statistically significant difference was found in the number of prosthetic joint infections in the group of surgeries with no antiplatelets or anticoagulants compared to surgeries with both medications administered during the 90 days preoperatively (0.82% vs 5.13%, *P*=0.0003). No significant difference was found between the groups with regard to stroke, myocardial infarction, pulmonary embolism, or deep venous thrombosis.

**Conclusion:** Surgeries for which both antiplatelets and anticoagulants were administered in the 90 days preoperatively had a statistically significantly higher rate of prosthetic joint infections compared to surgeries with neither medication administered preoperatively. Surgeons can use this information to better inform and risk-stratify patients prior to surgery.

## INTRODUCTION

Because of the aging population, the number of total hip and knee arthroplasties per year is estimated to reach 1.26 million and 635,000, respectively, by 2030.^1^ Mortality rates have been trending down despite comorbidities increasing for patients undergoing total joint replacements.^2^ Major nonorthopedic complications include cardiac events, stroke, and venous thromboembolism (VTE). Myocardial infarction (MI) has replaced pulmonary embolism (PE) as the leading cause of death following total hip arthroplasties, likely in part from the widespread use of thromboprophylaxis.^2,3^ Risk stratification and identification of modifiable and nonmodifiable risk factors are essential in a less healthy and aging population.^4^

Antiplatelets and anticoagulants are frequently used in this patient group. An estimated 6 million people worldwide require chronic antithrombotic agents.^5^ Anticoagulants work by inhibiting formation of fibrin clots and are effective in both arterial and venous thrombosis.^6^ Anticoagulants include medications such as heparin, warfarin, dabigatran, rivaroxaban, and apixaban. Indications for anticoagulant use include atrial fibrillation, mechanical heart valves, stroke prevention, and management or prevention of VTE or PE.^6^ Antiplatelets work to inhibit platelet aggregation and are primarily used for management of arterial disease.^6^ Two common antiplatelet agents are aspirin and clopidogrel. Common indications for antiplatelets are management of acute MI or revascularization procedures and prevention of MI and stroke. Antiplatelet agents and anticoagulants may increase bleeding risk and may lead to complications such as epidural hematoma, wound drainage, hematoma, and transfusion.^6^

The hypothesis of this study was that preoperative use of an anticoagulant or antiplatelet agent increases the risk of postoperative complication. This study aimed to determine if surgeries with anticoagulant and/or antiplatelet therapy administered within 90 days preoperatively had an increased risk of complications in a 30-day postoperative period compared to surgeries without either class of drugs administered preoperatively.

## METHODS

This project was submitted to and approved by the Ochsner Clinic Foundation Institutional Review Board prior to commencement. A retrospective medical record review was conducted for primary or revision total hip or knee arthroplasties from November 2012 through March 2016. Surgeries were queried using Current Procedural Terminology (CPT) codes. Inclusion criteria were patients >18 years old and having history of arthroplasty that was either primary total hip (CPT code 27447), primary total knee (CPT code 27130), revision total hip of both acetabular and femoral components (CPT code 27487), or revision total knee of both femoral and tibial components (CPT code 27134). Indications for surgery were arthritis, stage 2 of 2 prosthesis reimplantation after infection, or revision for mechanical issue (eg, loosening, dislocation, or malalignment). Surgeries were excluded if the indication for surgery was fracture or stage 1 of 2 for infection (eg, antibiotic spacer placement).

A total of 1,544 patients undergoing 1,726 primary or revision total knee arthroplasty (TKA) or total hip arthroplasty (THA) were included in this study. A total of 168 patients underwent more than 1 surgery. A total of 630 surgeries were THAs (274 surgeries in males, 356 surgeries in females); 1,096 surgeries were TKAs (395 surgeries in males, 701 surgeries in females); 1,523 cases were primary surgeries (527 THAs, 996 TKAs); and 203 were revision surgeries (103 THAs, 100 TKAs).

Analysis of the data was based on surgeries rather than individual patients. The surgeries were divided into 4 groups (Table 1). Group 1 consisted of 1,213 surgeries without antiplatelet or anticoagulant therapy administered within 90 days preoperatively. Group 2 had 224 surgeries with antiplatelet therapy (aspirin or clopidogrel) administered within 90 days preoperatively. Group 3 consisted of 211 surgeries with anticoagulation (low molecular weight heparin, unfractionated heparin, warfarin, dabigatran, rivaroxaban, or apixaban) administered within 90 days preoperatively. Group 4 consisted of 78 surgeries with both antiplatelets and anticoagulation administered within 90 days preoperatively.

**Table 1.** Preoperative Antiplatelet and/or Anticoagulant Groups, n=1,726

Group	Preoperative Medications	Number of Surgeries
1	Surgeries without any antiplatelet or anticoagulant therapy administered within 90 days preoperatively	1,213
2	Surgeries with antiplatelet therapy administered within 90 days preoperatively (aspirin or clopidogrel)	224
3	Surgeries with anticoagulant therapy administered within 90 days preoperatively (low molecular weight heparin, unfractionated heparin, warfarin, dabigatran, rivaroxaban, apixaban)	211
4	Surgeries with both antiplatelet and anticoagulant therapy administered within 90 days preoperatively	78

Complications examined included superficial wound complication, prosthetic joint infection, MI, PE, stroke, and deep venous thrombosis (DVT). We defined prosthetic joint infection according to the 2011 Musculoskeletal Infection Society (MSIS)*.*^7^ Under the MSIS criteria, a prosthetic joint infection is defined by 1 major criterion or 4 minor criteria (Table 2). Superficial wound complications included any patient who did not meet MSIS criteria and was treated with either (1) antibiotics without surgery (eg, cellulitis or superficial wound drainage) or (2) surgical irrigation and debridement for noninfectious etiology (eg, seroma or hematoma). Fractures were not evaluated as a complication. Preoperative comorbidities and indications for preoperative use of antiplatelet or anticoagulant agents were not collected.

**Table 2.** Definition of Prosthetic Joint Infection by 2011 Musculoskeletal Infection Society^7^

**Major Criteria**
1. There is a sinus tract communicating with the prosthesis.
2. A pathogen is isolated by culture from 2 or more separate tissue and fluid samples obtained from the prosthetic joint.
**Minor Criteria**
1. Elevated serum erythrocyte sedimentation rate and serum C-reactive protein concentration
2. Elevated synovial white blood cell count
3. Elevated synovial polymorphonuclear percentage
4. Presence of purulence in the affected joint
5. Isolation of a microorganism in 1 culture of periprosthetic tissue or fluid
6. Greater than 5 neutrophils per high-power field in 5 high-power fields observed from histologic analysis of periprosthetic tissue at × 400 magnification

Note: Prosthetic joint infection is defined by 1 major criterion or 4 minor criteria.

All patients were medically optimized preoperatively, and the surgeries were performed by 3 fellowship-trained surgeons at an academic urban tertiary referral center. The perioperative protocol was standardized among all groups. Preoperative antiplatelet and anticoagulant agents were typically held 4 days preoperatively without bridging anticoagulation. Bridging anticoagulation was used in cases only when recommended by a patient's cardiologist or hematologist. When possible, neuraxial anesthesia was used. Reasons for not receiving neuraxial anesthesia were inability to place a spinal epidural and cases in which antiplatelet and/or anticoagulant medications were not discontinued in the proper time frame and epidural hematoma formation was a concern. Preoperative antibiotics were 2 g of cefazolin and 1 g of vancomycin administered intravenously before incision. For patients with a documented adverse reaction to cephalosporins, 900 mg of clindamycin was typically administered instead. Postoperatively, patients received 3 doses of intravenous (IV) 2 g cefazolin, or for patients with adverse reaction, 3 doses of IV 900 mg clindamycin. Patients received 3 g of tranexamic acid in 100 mL of solution topically at the time of arthrotomy closure. All patients went through early goal-directed physical therapy, mechanical prophylaxis with sequential compression device, and aspirin 325 mg twice daily or dose-adjusted warfarin based on risk stratification. Patients who were taking warfarin preoperatively or had a personal or strong family history of VTE were started on warfarin with a goal international normalized ratio (INR) of 1.8 to 2.2. Patients were restarted on other oral antiplatelet and anticoagulant agents based on recommendations by their cardiologist or hematologist. Duration of therapy and drug selection in this group were dependent on medical history and preoperative medications.

Standard chi-square analysis was used to compare the 4 groups to each other (*P*<0.05 conferred significance). Fisher exact test was used to confirm statistical significance if indicated because of small population size. The Tukey honestly significant difference (HSD) post hoc test was used assuming these data met the homogeneity of variances assumption. This test was performed using 15 degrees of freedom for the error term and *P*=0.01 and *P*=0.05 in the range distribution for significance level.

## RESULTS

A total of 1,726 surgeries were included in this study. Superficial wound complications were the most common complication among all groups, occurring in 66 surgeries (3.82%) (Table 3). As shown in Table 4, no statistically significant difference in wound complications was found between group 1 compared to groups 2 through 4 (group 1 vs 2, *P*=0.667; group 1 vs 3, *P*=0.300; group 1 vs 4, *P*=0.951).

**Table 3.** Total Number and Percentage of Postoperative Complications

Group	Superficial Wound Complication	Prosthetic Joint Infection	Myocardial Infarction	Pulmonary Embolism	Deep Venous Thrombosis	Stroke
1, n=1,213	45 (3.71)	10 (0.82)	3 (0.25)	4 (0.33)	15 (1.24)	0 (0)
2, n=224	7 (3.13)	3 (1.34)	2 (0.89)	2 (0.89)	3 (1.34)	0 (0)
3, n=211	11 (5.21)	3 (1.42)	1 (0.47)	1 (0.47)	3 (1.42)	1 (0.47)
4, n=78	3 (3.85)	4 (5.13)	1 (1.28)	0 (0)	2 (2.56)	0 (0)
Total, n=1,726	66 (3.82)	20 (1.16)	7 (0.41)	7 (0.41)	23 (1.33)	1 (0.06)

Note: Data are presented as n (%).

**Table 4.** Postoperative Complication Comparisons

Group Comparison	Superficial Wound Complication	Prosthetic Joint Infection	Myocardial Infarction	Pulmonary Embolism	Deep Venous Thrombosis	Stroke
1 vs 2	0.667	0.455	0.132	0.230	0.899	–
1 vs 3	0.300	0.400	0.566	0.744	0.824	0.016^a^
1 vs 4	0.951	0.0003^b^	0.111	0.612	0.319	–

Note: Data are *P* values.

^a^Insignificant on Fisher analysis (*P*=0.148).

^b^Significant on Fisher analysis (*P*=0.0075).

Prosthetic joint infections occurred in 20 surgeries (1.16%), 10 of those surgeries (0.82%) from group 1 and 4 (5.13%) from group 4. This finding was statistically significant under chi-square testing (*P*=0.0003) and confirmed with Fisher analysis (*P*=0.0075). No statistical difference in prosthetic joint infections was found between the other groups (group 1 vs 2, *P*=0.455; group 1 vs 3, *P*=0.400).

Symptomatic DVT proven with ultrasound was found in a total of 23 surgeries (1.33%), 15 of those surgeries in group 1 (1.23%), but no statistically significant difference between the groups was found (group 1 vs 2, *P*=0.899; group 1 vs 3, *P=*0.824; group 1 vs 4, *P=*0.319).

No statistically significant differences between any of the groups with regard to stroke, MI, and PE were found. In comparing group 1 to groups 2 through 4, MI results were *P*=0.132, *P*=0.566, and *P*=0.111, respectively. In comparing group 1 to groups 2 through 4, PE results were *P*=0.230, *P*=0.744, and *P*=0.612, respectively. No surgeries in groups 1, 2, or 4 had strokes reported postoperatively. A significant difference was found in the comparison of group 1 vs 3 for postoperative stroke (*P*=0.016), but because of the small sample size, this result was insignificant on Fisher analysis (*P*=0.148).

A post hoc analysis using the Tukey HSD resulted in a range statistic (Q) of 4.37 to 5.56. This method strongly suggested that the difference between groups 1 vs 4 was statistically significant (Q=4.48).

## DISCUSSION

This study found that prosthetic joint infections occurred more frequently with surgeries in which both an antiplatelet and anticoagulant agent were administered within 90 days preoperatively (5.13%) compared with surgeries without either medication class prior (0.82%). This finding was statistically significant under chi-square testing (*P*=0.0003). Comparing these findings to the literature, a study by Rudasill et al^8^ evaluated 17,567 total hip arthroplasty patients with regard to preoperative INR and postoperative outcomes. Data were extracted from the National Surgical Quality Improvement Program database. Mortality increased from 0.3% in patients with INR <1.0 compared to 4.9% in patients with INR ≥1.5, independent of other comorbidities. The study also found increased length of stay and increased bleeding risk with increasing INR. A study by Zarling et al evaluated preoperative medications for 3,959 patients undergoing total joint arthroplasty.^9^ The study found 4.02% of THA and 5.03% of TKA patients received anticoagulants within 30 days preoperatively, while antiplatelet use was much higher at 42.03% (THA) and 44.14% (TKA). A statistically significant higher odds ratio (OR) for readmission with preoperative use of antiplatelets in THA patients (OR 2.25, *P*=0.004) and anticoagulants in TKA patients (OR 2.041, *P*=0.015) was found. This study did not find a statistically significant difference in discharge disposition (home vs extended-care facility) with antiplatelet or anticoagulant agent use.

With an increasing number of total joint arthroplasties being performed annually, the need for continued improvement in patient outcomes is obvious. Identification of preoperative risk factors allows for better medical optimization and is important for counseling patients regarding possible outcomes. The purpose of this study was to determine the relationship between preoperative antiplatelet or anticoagulant status and postoperative complications. This study did not find significant differences among the surgery groups when looking at rates of MI, PE, stroke, DVT, and superficial wound complications. However, significantly more surgeries were associated with prosthetic joint infections in the group taking both preoperative antiplatelets and anticoagulants vs the group taking neither. This finding makes sense logically, as the use of antiplatelet and anticoagulant agents typically implies certain comorbidities such as history of DVT or atrial fibrillation. As such, the use of these medications is likely an indicator of higher risk patients. The preoperative use of antiplatelets and anticoagulants should be part of the preoperative evaluation and counseling.

A particular strength of this study is the number of patients included at a single center with a standardized postoperative protocol. Additionally, the study was conducted based on review of actual medical records, not administrative data. A limitation of the study is that the comorbidities were not independently analyzed. Additionally, indications for anticoagulant or antiplatelet agent use were not collected; however, indications would likely correlate with patient comorbidities in the majority of cases. A confounding effect between preoperative use of an anticoagulant or antiplatelet agent and patient comorbidities was likely in this study. As such, the use of these medications may act as a surrogate for patient comorbidities and act as an easily identifiable risk factor. An analysis including comorbidities would help elucidate whether the findings of this study are simply the effect of confounding comorbidities or if anticoagulant or antiplatelet agent use is an independent risk factor for complications.

## CONCLUSION

Total knee or hip arthroplasty with preoperative use within 90 days of both antiplatelet and anticoagulant agents was associated with a significantly higher rate of prosthetic joint infections during the first 30 days after surgery compared to surgeries in which neither medication was administered preoperatively. No significant differences in superficial wound complications, MI, PE, DVT, or stroke were found. As the majority of patients are on antiplatelet and anticoagulant medications for nonmodifiable comorbidities, this information should be used when counseling, optimizing, and risk-stratifying patients prior to surgery.

## Notes

*This article meets the Accreditation Council for Graduate Medical Education and the American Board of Medical Specialties Maintenance of Certification competencies for Patient Care, Medical Knowledge, and Practice-Based Learning and Improvement.*
